# Adolescents’ Cyber Victimization: The Influence of Technologies, Gender, and Gender Stereotype Traits

**DOI:** 10.3390/ijerph17041293

**Published:** 2020-02-17

**Authors:** Michelle F. Wright, Sebastian Wachs

**Affiliations:** 1 Department of Psychology, Pennsylvania State University, State College, PA 16802, USA; mfw5215@psu.edu; 2 Faculty of Social Studies, Masaryk University, 60200 Brno, Czechia; 3 Department of Educational Studies, University of Potsdam, 14476 Potsdam, Germany

**Keywords:** gender, gender stereotype trait, cyber victimization, technology

## Abstract

The purpose of the present study was to investigate the role of gender and gender stereotype traits (masculinity, femininity) in cyber victimization behaviors (cyber relational victimization, cyber verbal victimization, hacking) through different technologies (mobile phones, gaming consoles, social networking sites). There were 456 8th graders (226 females; M age = 13.66, SD = 0.41) from two midwestern middle schools in the United States included in this study. They completed questionnaires on their endorsement of masculine and feminine traits, and self-reported cyber victimization through different technologies. The findings revealed main effects of types of cyber victimization for boys and of technology for girls. In particular, boys with feminine traits experienced the most victimization by cyber verbal aggression, cyber relational aggression, and hacking when compared to the other groups of boys. Girls with feminine traits experienced the most cyber victimization through social networking sites, gaming consoles, and mobile phones in comparison to the other groups of girls. For girls with feminine traits, they reported more cyber relational victimization and cyber verbal victimization through mobile phones and social networking sites, as well as more hacking via social networking sites. Such findings underscore the importance of considering gender stereotype traits, types of victimization, and technologies when examining cyber victimization.

## 1. Introduction

Cyber victimization is one consequence of technology use. Cyber victimization is defined as experiencing aggressive behaviors via information and communication technologies, such as the internet, gaming consoles, and mobile phones [1,2,3,4,5,6,7,8,9,10]. Research has focused on gender as a predictor of cyber victimization, but such a focus ignores the potential influence of contextual and cultural factors [11,12,13,14,15,16]. A more fruitful direction is to examine not only gender but also the impact of gender stereotype traits, defined as behaviors and attitudes that are acceptable, appropriate, or desirable for people based on biological sex, such as masculinity and femininity, on cyber victimization. To address this gap in the literature, the present study examined the joint roles of technology and victimization type in girls’ and boys’ experience of cyber victimization by also examining the influence of their gender and endorsement of masculine and feminine traits. Findings of this study add to the existing literature concerning gender differences in adolescents’ experience of cyber victimization by providing more details about the types of different victimization, technologies where victimization occurred, and gender stereotype traits.

### 1.1. Cyber Victimization and Gender

Unlike cyberbullying (i.e., experience of aggressive, intentional act carried out by a group or individual, using technologies, repeatedly and over time against a victim who cannot easily defend him or herself), cyber victimization does not necessarily include repetition or an imbalance of power [17,18]. Examples of cyber victimization include harassment, stalking, abuse, assault, hostility, happy slapping, outing, and flaming. Variations exist in the literature concerning the frequency of cyber victimization, from 4% to 43% depending on the study [19,20]. Despite these variations, which potentially reflect sampling and measurement differences, cyber victimization clearly occurs among adolescents. Cyber victimization relates to psychological functioning difficulties, such as school failure, usage of drugs or alcohol, and depression [21].

Gender has also been extensively examined as a variable which may provide insight into the characteristics that put adolescents at risk for cyber victimization. However, gender has proven to be an inconsistent predictor of adolescents’ risk of experiencing these behaviors as gender differences in cyber victimization are mixed. For instance, various researchers have found that boys were more likely to be victims of cyberbullying and cyber aggression [22,23], whereas others have found that girls experienced more types of victimization when compared to boys [20,24]. On the other hand, some researchers have found no gender differences in cyber victimization [25,26,27].

To account for these conflicting findings, Underwood and Rosen [8] appealed for researchers “to move beyond examining mean level gender differences in cyberbullying” (p. 12). Instead of mean level gender differences, they proposed that researchers should focus on the methods (i.e., technologies where victimization is experienced) and mechanisms (i.e., types of cyber victimization) through which girls and boys are harmed via the cyber context. In a recent study, Görzig and Frumkin [28] investigated cyber victimization through mobile devices among 1300 participants aged 9–16 across various European countries. Findings indicated that girls were more likely to be victimized through mobile devices when compared to boys. These results replicated those from an earlier study with Spanish participants aged 12–17 [10]. However, no gender differences in text messaging and mobile phone victimization were found among children and adolescents aged 11–18 in New Zealand [29] and 10–18 years old in Belgium [30]. Focusing on specific types of victimization rather than technological mediums, Popovic-Citic and colleagues [31] found that Serbian boys were more likely to experience harassment, denigration, and outing through the cyber context. In one of the few studies to examine both types of victimization and technologies where victimization occurred, Dehue and colleagues [9] asked 1211 adolescents (M age = 12.7, 50.5% boys) if they had been bullied via Microsoft Service Network (MSN) messenger or email, and whether they were harmed by hacking, ignoring, name-calling, and gossiping behaviors. Findings indicated that girls were more likely to be victimized via MSN messenger and by hacking, name-calling, gossiping, and blaming behavior. Although Dehue et al. [9] provided a good foundation for future research, little attention has been given to whether certain types of cyber victimization were more common through specific technologies. Furthermore, the focus on gender in these previous studies does not necessarily indicate the level of masculine and feminine traits that adolescents endorse. A focus on these traits and gender might provide a better understanding of adolescents’ risk of experiencing cyber victimization.

### 1.2. Masculine Traits, Feminine Traits, and Aggression

Terman and Miles [32] developed a theoretical foundation for understanding masculinity and femininity by conceptualizing various traits which could distinguish males and females. This conceptualization was informed by ideal traits, interests, and attitudes that were associated with biological sex. Such a framework hypothesized that gender as a single bi-polar dimension, with masculinity and femininity on opposite ends. Instrumental personality characteristics, including individualism, self-affirmation, risk-taking, social dominance, and aggressiveness, make up the dimension of masculinity [33]. In contrast, expressive traits, such as warmth, sensitivity, and altruism, represent the dimension of femininity. Someone’s gender role orientation is determined by the extent to which him or her endorse masculine or feminine norms [34]. Through these norms, males learn that aggression reinforces and maintains the masculine role, while females learn to inhibit aggressive behaviors or to engage in covert forms of aggressive behaviors to better align with the feminine role [35]. As a consequence of these gender schemas, the justification of certain behaviors as being masculine or feminine might be maintained and reinforced in adolescents’ peer relationships.

Victimization is associated with feminine traits among adolescent females and males [36]. It is currently unknown how masculine and feminine traits relate to cyber victimization. As some forms of cyber victimization, such as being targeted by rumors and relationship manipulation, are conceptualized as forms of relational aggression, it might be expected that feminine traits predict the involvement in these behaviors. In addition, physical forms of cyber victimization, such as hacking, may be associated with masculine traits.

### 1.3. The Present Study

Based on the literature, the aim of this study was to clarify the inconsistent findings concerning gender differences in adolescents’ experiences of cyber victimization by considering the joint role of type of cyber victimization, technology where victimization occurred, and the endorsement of masculine and feminine traits. In the present study, cyber victimization included cyber relational aggression (i.e., gossiping, rumor spreading), cyber verbal aggression (i.e., saying mean things, teasing, insulting), and hacking. Technologies included social networking sites (SNS; e.g., Facebook, Myspace, Twitter), gaming consoles (e.g., Xbox, PlayStation), and mobile phones (i.e., internet via these devices, text messages). Therefore, the present study aims to test what role gender and gender stereotype traits have in the interaction between cyber victimization and technology.

## 2. Method

### 2.1. Participants

There were 456 8th graders (226 females; M age = 13.66, SD = 0.41) from two suburban midwestern middle schools in the United States included in this study. Most adolescents self-identified as Caucasian (65.6%), followed by Latino/a (17.8%), Asian (8.2%), Black/African American (7.8%), Native Hawaiian (0.4%), and American Indian (0.2%). The schools were located in predominantly middle and upper socioeconomic neighborhoods. Parents reported a median income of $69,111.86, and most (86%) had at least a two-year college degree. G*Power was conducted to determine the minimum sample size, with a medium effect size. With an alpha = 0.05 and power = 0.80, the projected sample size was approximately 23 per group. The smallest group size was 26 in our study, indicating that there was more than adequate power to examine the study’s objectives.

### 2.2. Procedures and Measures

The study was approved by the university’s ethics board. Four schools were recruited for inclusion in the study, but two schools responded positively to the invite. The principal investigator met with school principals and teachers to introduce the study to them, explain how adolescents could participate, and what adolescents would be expected to do. On the same day, classroom announcements informing adolescents about how they could participate, what they would be expected to do if they participated, and the confidentiality of their answers were made to their homerooms. There were 565 parental permission slips distributed to adolescents. Of these, 495 parents/guardians agreed to allow their child to participate, 13 declined, and 57 slips were unreturned. Due to being absent on the day of data collection and the make-up days, 39 students were not included in the study, despite having parental permission. The final sample was 456 adolescents.

During data collection, adolescents provided their own assent to participate in the study. None declined to participate. They completed questionnaires on their background information (i.e., age, gender, ethnicity), masculine and feminine traits, and self-reported cyber victimization. Parents also provided demographic information, including their age, gender, education, and family income, which was sent back with the parental permission slip. Of the requested demographic information, 386 parents returned the survey. Regardless of whether parents sent back the demographic information survey, adolescents were still included in the study.

**Masculine and feminine characteristics.** To assess the extent to which adolescents identified with culturally prescribed traits regarding masculinity and femininity, the Bem Sex Role Inventory was administered [37]. There were 40 items, which were rated on a 7-point Likert scale (1 = *never* to 7 = *always*) concerning stereotypically masculine traits (e.g., dominant, selfish, independent) and stereotypically feminine traits (e.g., affectionate, gentle, compassionate). The items for masculine and feminine traits were averaged separately, creating separate scores on each of these traits. Cronbach’s alpha for masculine traits was 0.80 and 0.81 for feminine traits. Adolescents were placed in 1 of 4 groups based on how much they endorsed masculine and feminine traits. Groups were determined based on whether adolescents’ scores on masculine traits and feminine traits were above or below the median for each trait (for girls: M = 2.66 for masculine traits and M = 2.86 for feminine traits; for boys: M = 3.15 for masculine traits and M = 2.53 for feminine traits). Group 1 (boys and girls who endorsed both traits) included those adolescents who were high on both gender stereotype traits (*n* = 26 for girls; *n* = 29 for boys), high on masculine traits but low feminine traits for group 2 (boys and girls who endorsed masculine traits only; *n* = 48 for girls; *n* = 69 for boys), low masculine traits but high feminine traits for group 3 (boys and girls who endorsed feminine traits only; *n* = 60 for girls; *n* = 56 for boys), and group 4 included those adolescents who were low on both gender traits (boys and girls who endorsed low masculine and feminine traits; *n* = 92 for girls; *n* = 76 for boys).

**Self-reported cyber victimization.** Adolescents reported how often they experienced relational aggression (i.e., had rumors spread about themselves, being the target of gossip), verbal aggression (i.e., was insulted by someone, called mean names, teased in a mean way), and had someone log into their online account(s) without permission (i.e., hacking) through SNS, console gaming, and mobile phones [7]. There were fourteen items for relational aggression (seven items for rumor spreading and seven items for gossiping), twenty-one for verbal aggression (seven items for insulting someone, seven items for calling other means names, and seven items for teasing someone in a mean way), and seven for hacking, which were asked across the three different technologies where victimization occurred (e.g., 14 items for relational aggression multiplied by three technologies). For each victimization type, there were 42 items for cyber relational aggression (14 per technology type), 63 for cyber verbal aggression (21 per technology type), and 21 for hacking (7 per technology type), with a final total of 126 items for cyber victimization. Sample items included: How often have you spread rumors about a peer to make others not like him/her through social networking websites (relational aggression); how often have you called other peers mean names through social networking websites (verbal aggression); and how often have you logged into a peer’s account without his/her permission (hacking). Items were rated on a scale of 1 (*Never*) to 5 (*All of the Time*) and adolescents considered victimization within the current school year. Table 1 lists the Cronbach’s alphas for all types of victimization and technologies where victimized occurred. Each demonstrated adequate reliabilities (*αs* > 0.80).

## 3. Results

We examined the assumptions of a repeated-measures MANOVA before conducting the analyses. The data satisfied the first assumption by having continuous, dependent variables. The second assumption was satisfied by having related groups with at least two categories. The power analysis revealed that we needed a minimum of 23 participants per group and our lowest group number was 26, indicating that we satisfied assumption three by having adequate sample size. The data had no outliers, satisfying assumption four. We tested multivariate normality by conducting the Shapiro-Wilk test of normality, which indicated multivariate normality, satisfying assumption five. We plotted a scatterplot matrix and found a linear relationship for each group on the dependent variable, satisfying assumption six. Multicollinearity was not a problem for our analyses, with Variance Inflation Factors (VIF) under 1.81, which is less than a VIF of 10, indicating we satisfied assumption seven. A three-way repeated-measure MANOVA was conducted with gender and group as between-subject variables and with types of victimization and technologies where victimization occurred as within subject variables (see Table 2 for means and standard deviations).

Main effects of types of victimization (Wilks’ Λ = 0.97, *F* = 2.45, *p* < 0.05) and technologies where victimization occurred (Wilks’ Λ = 0.94, *F* = 3.06, *p* < 0.01) were found. A two-way interaction was found between type of behavior and technology where victimization occurred, Wilks’ Λ = 0.83, *F*(24,450) = 2.46, *p* < 0.001. Three-way interactions among types of victimization, technologies where victimization occurred, and gender (Wilks’ Λ = 0.91, *F*(24,448) = 1.93, *p* < 0.01) were found along with an interaction among types of victimization, technologies, and gender traits (Wilks’ Λ = 0.64, *F*(72,868) = 1.93, *p* < 0.001). The four-way interaction among types of victimization, technologies, gender, and gender traits was significant as well, Wilks’ Λ = 0.78, *F*(72,868) = 1.35, *p* < 0.05. Therefore, analyses were split by gender and then conducted again. For boys, there was a main effect of types of victimization (Wilks’ Λ = 0.97, *F* = 4.41, *p* < 0.01). For girls, main effects of technologies where victimization occurred (Wilks’ Λ = 0.92, *F* = 2.16, *p* < 0.05) were found, along with a two-way interaction between types of victimization and technologies (Wilks’ Λ = 0.77, *F*(21,224) = 1.80, *p* < 0.05), and a three-way interaction (Wilks’ Λ = 0.47, *F*(63,437) = 1.75, *p* < 0.001) among types of victimization, technologies, and gender traits.

The following explanation involves comparison of all four groups on each type of victimization and technology where victimization occurred, separated for boys and girls. Bonferroni corrections were used to examine differences between the groups. Boys who endorsed feminine traits experienced more cyber verbal aggression, cyber relational aggression, and hacking than the other groups of boys. In addition, boys who endorsed low feminine and masculine traits were victimized by cyber verbal aggression, cyber relational aggression, and hacking when compared to boys who endorsed both traits and boys who endorsed masculine traits only. There were no differences found for cyber victimization among boys who endorsed both traits and boys who endorsed masculine traits.

For girls, differences involved technologies where victimization occurred. Girls who endorsed feminine traits were the most targeted through all technologies (i.e., SNS, mobile phones, gaming consoles), followed by girls who endorsed low levels of masculine and feminine traits, and then girls who endorse masculine traits and girls who endorsed high levels of both traits. There was no significant difference between girls who endorsed masculine traits and those who endorsed both traits, and they experienced the lowest victimization through different technologies. Concerning the significant interaction between type of victimization and technologies where victimized occurred, girls who endorsed feminine traits were targeted more by cyber relational aggression and cyber verbal aggression through mobile phones and SNS than other groups of girls. In addition, girls who endorsed feminine traits also experienced more hacking via SNS.

## 4. Discussion

Research on gender differences in adolescents’ experience of cyber victimization is mixed. Despite these mixed findings, little attention has been given to understanding the combined role of types of cyber victimization and technologies where victimization occurred, and whether such considerations might shed more light on how gender might impact adolescents’ experience of cyber victimization. Such a focus is a more fruitful direction in understanding how gender might impact adolescents’ risk of cyber victimization. The purpose of the present study was to examine gender differences in cyber victimization by considering the joint role of types of cyber victimization (i.e., cyber relational victimization, cyber verbal victimization, hacking) and technologies where victimization occurred (i.e., SNS, console gaming, mobile phones). The present study also focused on the role of gender stereotype traits (i.e., masculinity, femininity).

The findings of the present study were complex, revealing that understanding types of cyber victimization provided a better understanding about the risk of cyber victimization for boys and their gender role endorsement. However, for girls and their gender role endorsement, considering the type of technologies where victimization occurred was important for understanding their risk of cyber victimization. For girls who endorsed feminine traits, their risk of cyber victimization was best understood through the types of cyber victimization and the technologies where victimization occurred.

### 4.1. Cyber Victimization, Gender, and Gender Traits

Boys who endorsed feminine traits experienced more cyber victimization via cyber relational aggression, cyber verbal aggression, and hacking, followed by the other groups of boys. Such findings suggest that boys who endorsed feminine traits were more frequently targeted for cyber victimization, which is supported by previous research on face-to-face victimization [36]. Similarly, boys who endorsed low masculine and feminine traits experienced more cyber relational aggression, cyber verbal aggression, and hacking than boys who endorsed high levels of these traits and those who endorsed masculine traits only. Boys who endorsed low levels of these traits experienced heightened levels of face-to-face victimization, and findings from the current research suggest that they also experience high levels of cyber victimization [38]. Both boys who endorsed masculine traits and those who endorsed both traits experienced the least cyber victimization, which is supported by previous literature indicating that they self-report lower face-to-face victimization [39].

Girls who endorsed feminine traits experienced the greatest cyber victimization through SNS, mobile phones, and gaming consoles when compared to the other girls, with girls who endorsed low levels of feminine and masculine traits self-reporting greater cyber victimization through these technologies than girls who endorsed masculine traits only and those who endorsed high levels of both traits. In addition, girls who endorsed both masculine and feminine traits and who endorsed masculine traits only experienced the least victimization through SNS, mobile phones, and gaming consoles, which is consistent with previous literature concerning face-to-face victimization [36,39]. Further supported by previous research is the finding that girls who endorsed feminine traits only and those who endorsed low levels of both traits were victimized more frequently through SNS, mobile phones, and gaming consoles when compared to girls who endorsed masculine traits only or both traits [38,39]. An interaction between type of cyber victimization and technologies where victimization occurred was found for girls who endorsed feminine traits such that they experienced more cyber relational aggression and cyber verbal aggression through SNS and mobile phones. They also experienced more hacking via SNS.

As indicated at the beginning of this section, comparing girls and boys on cyber victimization is difficult as separate main effects of types of cyber victimization and technologies where victimization occurred were found. However, there are some overarching conclusions that can be made. In particular, similar patterns were found such that the highest victimization was experienced by individuals who endorsed feminine traits and low levels of masculine and feminine traits, with the lowest victimization reported by individuals who endorsed masculine traits only or high levels of both traits [36,38,39]. These findings suggest the importance of not only understanding the effect of gender on cyber victimization, but gender stereotype traits as well. Although our study’s intent was to understand more about the complex relationship between gender and cyber victimization, we also found that considering gender stereotype trait endorsement further complicates this relationship but does provide a little bit more clarity about the risk of experiencing cyber victimization. Overall, it is clear that gender stereotype traits should be considered in studies of gender and cyber victimization based on the present study’s findings.

### 4.2. Future Directions and Limitations

The present study relied on a concurrent research design, which makes it difficult to identify changes in adolescents’ experience of cyber victimization. Future research should examine adolescents’ experience of different types of cyber victimization through various technologies by focusing on these variables among younger and older samples of adolescents. This research should also consider the potential for developmental changes to occur in the experience of various types of cyber victimization overtime. Such research should also consider that preferred technologies might change over time as well. The study involved self-report measures of cyber victimization. A fruitful direction in this research might be to include other informants of cyber victimization, such as peers, which has been demonstrated in previous research [8]. There were only two schools included in this study and the findings might not generalize to other adolescent samples. More research should be conducted with a larger sample to better understand the role of gender and gender roles in cyber victimization.

## 5. Conclusions

In summary, adolescents who endorsed more feminine traits and those who endorsed low levels of masculine and feminine traits reported the highest levels of cyber victimization. The lowest cyber victimization was reported by adolescents who endorsed masculine traits only or had high endorsement on both traits. Along with gender, gender stereotype traits further our understanding of adolescents’ experience of different types of cyber victimization and the technologies where victimization occurred. In particular, for boys, main effects of type of victimization were found, while an interaction between type of victimization and technologies were found for girls. Such a finding highlights the importance of delineating different types of cyber victimization and technologies where victimization occurred to fully understand adolescents’ experience of cyber victimization. Furthermore, the findings indicate a need for school-based interventions on reducing cyber victimization. One such promising intervention is the Tutoría Entre Iguales (TEI program) designed to increase school climate and reduce cyber victimization [40]. Implementing the TEI program and other effective programs are important for “battling” cyber victimization among adolescents.

## Figures and Tables

**Table 1:** Cronbach’s alphas of all victimization behaviors and technologies.

Victimization Type and Technology	Cronbach’s Alpha	Number of Items
Cyber Relational Aggression		42 in total
Social Networking Sites	0.95	14
Console Gaming	0.92	14
Mobile Phones	0.93	14
Cyber Verbal Aggression		63 in total
Social Networking Sites	0.88	21
Console Gaming	0.91	21
Mobile Phones	0.90	21
Hacking		21 in total
Social Networking Sites	0.93	7
Console Gaming	0.80	7
Mobile Phones	0.83	7

**Table 2:** Means and standard deviations for gender and groups based on gender stereotype traits based on cyber victimization.

Type of Victimization and Behavior	Group 1 (*n* = 55)		Group 2 (*n* = 117)		Group 3 (*n* = 116)		Group 4 (*n* = 168)		Significant Group Differences
	Boys M (SD)	Girls M (SD)	Boys M (SD)	Girls M (SD)	Boys M (SD)	Girls M (SD)	Boys M (SD)	Girls M (SD)	
CRA	2.54 (1.43)	2.61 (1.53)	2.55 (1.44)	2.63 (1.47)	2.73 (1.51)	2.59 (1.41)	2.60 (1.43)	2.59 (1.43)	Boys: Group 3 > Group 4, Group 1, and Group 2
									Boys: Group 4 > Group 1 and Group 2.
									No differences for girls.
CVA	2.76 (1.21)	2.73 (1.36)	2.79 (1.27)	2.74 (1.37)	2.97 (1.03)	2.75 (1.01)	2.83 (1.36)	2.74 (1.24)	Boys: Group 3 > Group 4, Group 1, and Group 2
									Boys: Group 4 > Group 1 and Group 2
									No differences for girls.
Hacking	1.69 (1.01)	1.70 (1.00)	1.67 (1.06)	1.66 (.99)	1.89 (1.00)	1.73 (1.03)	1.70 (.89)	1.71 (1.05)	Boys: Group 3 > Group 4, Group 1, and Group 2
									Boys: Group 4 > Group 1 and Group 2
									No differences for girls.
SNS	2.53 (1.43)	2.56 (1.47)	2.56 (1.43)	2.57 (1.45)	2.59 (1.36)	2.81 (1.47)	2.62 (1.36)	2.63 (1.51)	Girls: Group 3 > Group 4, Group 1, and Group 2
									Girls: Group 4 > Group 1 and Group 2
									No differences for boys.
Mobile Phones	2.75 (1.30)	2.78 (1.31)	2.76 (1.36)	2.78 (1.19)	2.79 (1.11)	3.00 (1.03)	2.80 (1.41)	3.83 (1.39)	Girls: Group 3 > Group 4, Group 1, and Group 2
									Girls: Group 4 > Group 1 and Group 2
									No differences for boys.
Gaming Consoles	1.61 (1.01)	1.63 (1.08)	1.65 (1.04)	1.67 (1.03)	1.69 (1.01)	1.92 (1.03)	1.70 (1.08)	1.72 (.93)	Girls: Group 3 > Group 4, Group 1, and Group 2
									Girls: Group 4 > Group 1 and Group 2
									No differences for boys.

Note. All items were rated on a scale of 1 (never) to 5 (all of the time). CRA = cyber relational victimization; CVA = cyber verbal victimization; SNS = social networking sites. Group 1 included boys and girls who endorsed both masculine and feminine traits (androgynous), Group 2 included boys and girls who endorsed masculine traits only, Group 3 included boys and girls who endorsed feminine traits only, and Group 4 included boys and girls who were low on both masculine and feminine traits (undifferentiated).
